# An atlas of anatomical variants of subsegmental pulmonary arteries and recognition error analysis

**DOI:** 10.3389/fonc.2023.1127138

**Published:** 2023-03-13

**Authors:** Hao Xu, Heng Zhao, Jian Jin, Jiayi Geng, Chao Sun, Dawei Wang, Nan Hong, Fan Yang, Xiuyuan Chen

**Affiliations:** ^1^ Department of Thoracic Surgery, Peking University People’s Hospital, Beijing, China; ^2^ Thoracic Oncology Institute, Peking University People’s Hospital, Beijing, China; ^3^ Department of Radiology, Peking University People’s Hospital, Beijing, China; ^4^ Institute of Advanced Research, Infervision Medical Technology Co., Ltd, Beijing, China

**Keywords:** pulmonary artery, lung variation, 3D reconstruction, precision surgery, artificial intelligence

## Abstract

**Background:**

Surgery, including lobectomy and segmentectomy, is the major curative intervention for lung cancer. Surgical planning for pulmonary surgery is difficult due to the high variation rate of pulmonary arteries and needs a fine-grained atlas as a reference. We conducted a study to create a surgically oriented atlas and analyzed the error encountered during the production.

**Method:**

A total of 100 Chest CTs performed at Peking University People’s Hospital from 2013.09 to 2020.10 were randomly selected for segmental artery labeling. Dicom files were collected for 3D reconstruction. Manual segmentation of each segmental artery was performed by 4 thoracic surgeons. Cross-validation by surgeons was performed to establish the golden standard based on their consensus. Initial recognition errors were recorded accordingly.

**Result:**

The most frequently seen variants for the right upper lobe is 2-branch RA^1^+^2^rec+^3^ and RA^2^asc; right middle lobe 2-branch RA^4^a and RA^4^b+^5^; right lower lobe 3-branch RA^7^, RA^8^ and RA^9^+^10^; left upper lobe 3-branch LA^1+2^a+^3^, LA^1+2^b, LA^1+2^c and 1-branch LA^4^+^5^; left lower lobe 2-branch LA^8^ and LA^9^+^10^. Top 5 segmental error occurs in RA^4^ (23%), LA^8^ (17%), RA^9^ (17%), RA^8^ (14%) and LA^9^ (11%). A rapid surgical planning tool form was created based on high frequency anatomic variants.

**Conclusion:**

Our research provided an atlas for lobectomy and segmentectomy at the subsegmental or more distal level. We demonstrated that the recognition accuracy of pulmonary arteries in a non-time-sensitive experimental scenario was still unfavorable. We also suggest that extra attention should be paid to certain surgeries during the surgical planning process.

## Background

Lung cancer carries the highest mortality among all malignancies worldwide (1), for which surgery, including anatomic lobectomy and segmentectomy, is by far the best curative treatment (2–4).

Delicate anatomic pulmonary resection requires a complex preoperative planning process that consists of 3 steps: 3-dimensional reconstruction, anatomic variant recognition, and intra-operative imaging projection (5). Since segmental arteries of the lung are known for abundancy in anatomic variations, of which the misidentification may lead to catastrophic consequences, the identification of the anatomic variant is the core of the whole planning process. However, the surgical planning process of surgeons is abstract and difficult to visualize or standardize, which prevents the process from being evaluated.

For the purpose of improving the surgical planning skill of thoracic surgeons, multiple anatomic atlases of pulmonary arteries were created. Traditional atlas has given us the panorama of subsegmental variants. However, in the era of precision surgery, more detailed information may be necessary. Artery variant recognition is crucial for segmentectomy and lobectomy planning at the subsegmental or even more distal level. The ignorance of even an anonymous small artery that branches directly from the main trunk pulmonary artery may lead to massive intra-operative hemorrhage. Thus, a modern atlas that is more precision surgery-oriented is in need.

To accomplish an atlas on such detail, new methodologies are mandatory. Three-dimensional (3-D) reconstruction tool has been utilized in various surgical planning processes with the time efficiency as its primary pitfall (6). Thanks to the emergence of automated 3-D reconstruction algorithms (5, 7–10), we are able to visualize anatomic variants in a more intuitive way. We hereby present a comprehensive pulmonary artery atlas to aid lobectomy and segmentectomy. We also assessed the accuracy of the identification of each artery and present a convenient tool for surgery planning.

## Method

### Patient enrollment

We randomly selected 100 patients who underwent chest computed tomography (CT) at Peking University People’s Hospital from 2013.09 to 2020.10. The inclusion criteria were as follows: (1) thin-section (≤ 1.5 mm), either non-enhanced chest CT images or contrast-enhanced chest CT images available, and (2) 3-D reconstruction of pulmonary vessels and bronchi available.

### Chest CT Acquisition and 3-D reconstruction

The chest CT was scanned from the thoracic entrance to the bottom of the lung after one inhalation and holding breath using CT instruments from GE Healthcare (Chicago, Ill, USA), Philips Healthcare (Amsterdam, Netherlands), and Siemens Healthineers. All dicom files were de-identified. Reconstructions were performed for all CT images using the automated 3-D reconstruction system (InferOperate Thoracic Surgery) (6) of pulmonary blood vessels and bronchi, and STL files were exported for labeling use. All 3-D models were reviewed to ensure a fine-grained demonstration of pulmonary arteries to subsegmental or, if necessary, more distal level.

### Image labeling and golden standard establishment

**Figure 1:**
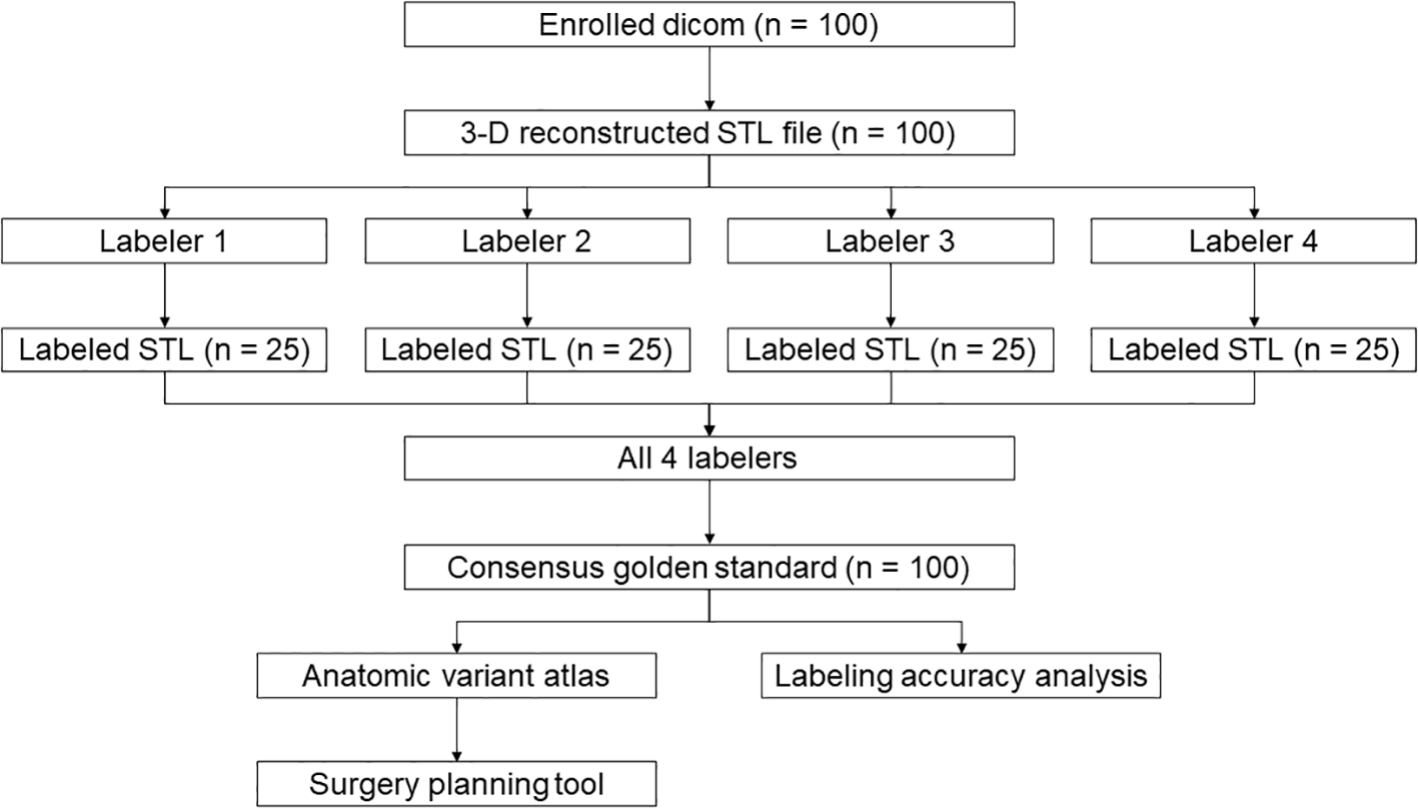
Study design.

All CTs and corresponding 3-D reconstructions were equally divided into 4 sub-cohorts that were assigned to 4 senior residents or attendings specialized in thoracic surgery for labeling. The labeling process was as follows: The artery of each segment was recognized to the necessary level no more distant than sub-subsegmental branches; each branch of the subsegmental or more distal artery was segmented on the 3D model and then merged into one single STL file under the name of the segment (i.e., RA1.stl). The artery of each segment was segmented and labeled using Materialise Mimics (Materialise NV, Leuven, Belgium). Each labeling surgeon was responsible for 25 cases. To generate the golden standard, each labeled reconstruction model was cross-validated by 3 other surgeons. The golden standard was established based on the consensus of all 4 participating surgeons. The recognition accuracy of surgeons was evaluated by comparison with the golden standard (**Figure 1**).

## Result

### Baseline characteristics

All CTs were reconstructed and labeled successfully. The median age of all patients was 51(IQR 40-59) years, and 52 (52%) patients were male. The slice thickness of all CTs was less than or equal to 1.5 mm, of which 1.0 mm slice thickness was mostly seen (47%), followed by 1.5 mm (33%) and 1.25 mm (20%) (**Table 1**). The anatomic variant of each lobe was depicted respectively as follows.

**Table 1:** Baseline information of materials.

Characteristics	No.(%)/Median(IQR)
Age, years	51(40-59)
Gender	
Male	52(52%)
Female	48(48%)
Slice thickness	
1mm	47(47%)
1.25mm	20(20%)
1.5mm	33(33%)
Machine	
GE	19(19%)
Philips	8(8%)
SIEMENS	64(64%)
TOSHIBA	8(8%)
UIH	1(1%)

UIH, United Imaging Healthcare Surgical Technology Co., Ltd.

### Anatomical variant of the right upper lobe

The two-branch type of right upper lobe arteries was observed in 63% of all patients, which was the most common type. Eighteen percent of patients had a single stem, and the three-branch type was observed in 19% of patients.

**Figure 2:**
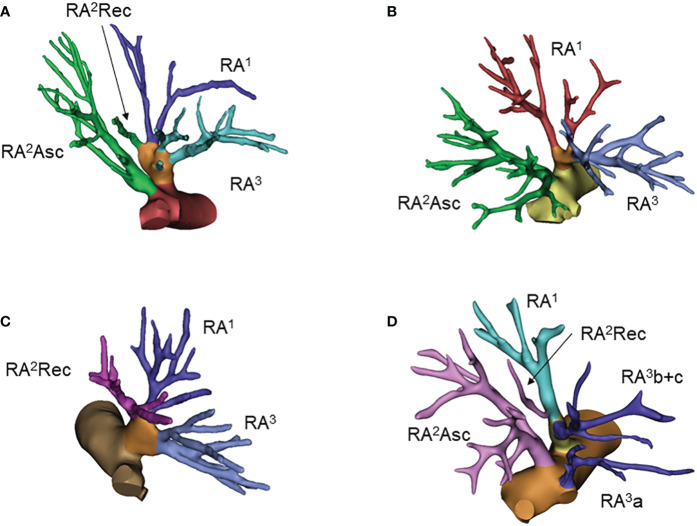
Common anatomic variants of the right upper lobe. **(A)** RA^1^+^2^rec+^3^, RA^2^asc; **(B)** RA^1^+^3^, RA^2^asc; **(C)** RA^1^+^2^rec+^3^; **(D)** RA^1^+^2^rec+^3^b, RA^2^asc, RA^3^a.

In 39% of patients, the right upper arteries bifurcated into RA^1^+^2^rec+^3^ and RA^2^asc, which was the most common pattern. The rate of branching pattern of the RA^1^+^2^rec+^3^ type and RA^1^+^3^ and RA^2^asc type were both 18%, tied for the second most common variant. Frequencies of remaining patterns of RA^1^+^2^rec+^3^b, RA^2^asc and RA^3^a type, RA^1^+^2^rec+^3^b and RA^3^a type, RA^1^+^2^rec+^3^a, RA^2^asc and RA^3^b type, RA^1^+^3^b, RA^2^asc and RA^3^a type, RA^1^+^3^a, RA^2^asc and RA^3^b type, and RA^1^+^2^rec+^3^ai+b and RA^2^asc+^3^aii type were 8%, 4%, 4%, 4%, 3% and 2%, respectively (**Table 2**). Anatomic variants with frequencies higher than 5% were visualized in **Figure 2**; **Supplementary Figure 1**.

**Table 2:** Right upper lobe.

Artery variants	Abbreviation	Frequency
RA^1^+^2^rec+^3^	1+2+3	18%
RA^1^+^2^rec+^3^, RA^2^asc	1+2+3, 2	39%
RA^1^+^2^rec+^3^b, RA^3^a	1+2+3, 3	4%
RA^1^+^2^rec+^3^ai+b, RA^2^asc+^3^aii	1+2+3, 2 + 3	2%
RA^1^+^3^, RA^2^asc	1+3, 2	18%
RA^1^+^2^rec+^3^b, RA^2^asc, RA^3^a	1+2+3, 2, 3	8%
RA^1^+^2^rec+^3^a, RA^2^asc, RA^3^b	1+2+3, 2, 3	4%
RA^1^+^3^b, RA^2^asc, RA^3^a	1+3, 2, 3	4%
RA^1^+^3^a, RA^2^asc, RA^3^b	1+3, 2, 3	3%

rec, recurrent; asc, ascending.

### Anatomical variant of the right middle lobe

The two-branch type was the most common pattern of right middle lobe arteries, which was observed in 75% of all patients. The single stem as RA^4^+^5^ was the second most common pattern, accounting for 21%.

**Figure 3:**
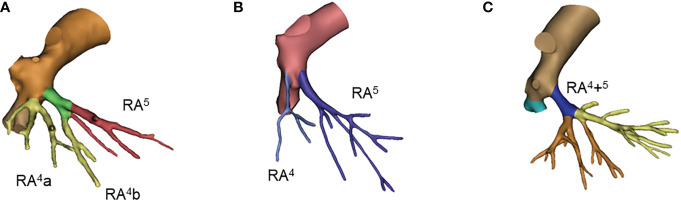
Common anatomic variants of right middle lobe. **(A)** RA^4^a+RA^4^b+^5;^ **(B)** RA^4^, RA^5^; **(C)** RA^4^+^5^.

For the right middle lobe, the most common bifurcated types were RA^4^a and RA^4^b+^5^, and RA^4^ and RA^5^ with percentages of 41%, 31%. Single stem RA^4^+^5^ was seen in 21% of cases (**Figure 3**; **Supplementary Figure 2**). The remaining types accounted for only 7 percent in total. Interestingly, cross-lobular variation was seen in 4 patients, and the branches of the middle lobe artery originated from the basilar artery, namely, RA^4^a (1%), RA^4^b (2%) and RA^5^bi (1%) (**Table 3**).

**Table 3:** Right middle lobe.

Artery variants	Abbreviation	Frequency
RA^4^+^5^	4+5	21%
RA^4^+^5^b, RA^5^a	4+5, 5	1%
RA^4^+^5^a, RA^5^b	4+5, 5	1%
RA^4^a, RA^4^b+^5^	4, 4 + 5	41%
RA^4^, RA^5^	4, 5	31%
RA^4^a+bi, RA^5^	4, 5	1%
RA^4^ai+^5^, RA^4^aii, RA^4^bi	4+5, 4, 4	1%
RA^4^a, RA^4^b, RA^5^	4, 4, 5	1%
RA^4^ai, RA^4^aii, RA^4^b+^5^	4, 4, 4 + 5	1%
RA^4^, RA^5^a+bi, RA^5^bii	4, 5, 5	1%
RML artery branches from BPA		4%
RA^4^b		2%
RA^4^ai		1%
RA^5^bi		1%

BPA, basal pulmonary artery.

### Anatomical variant of the right lower lobe

**Figure 4:**
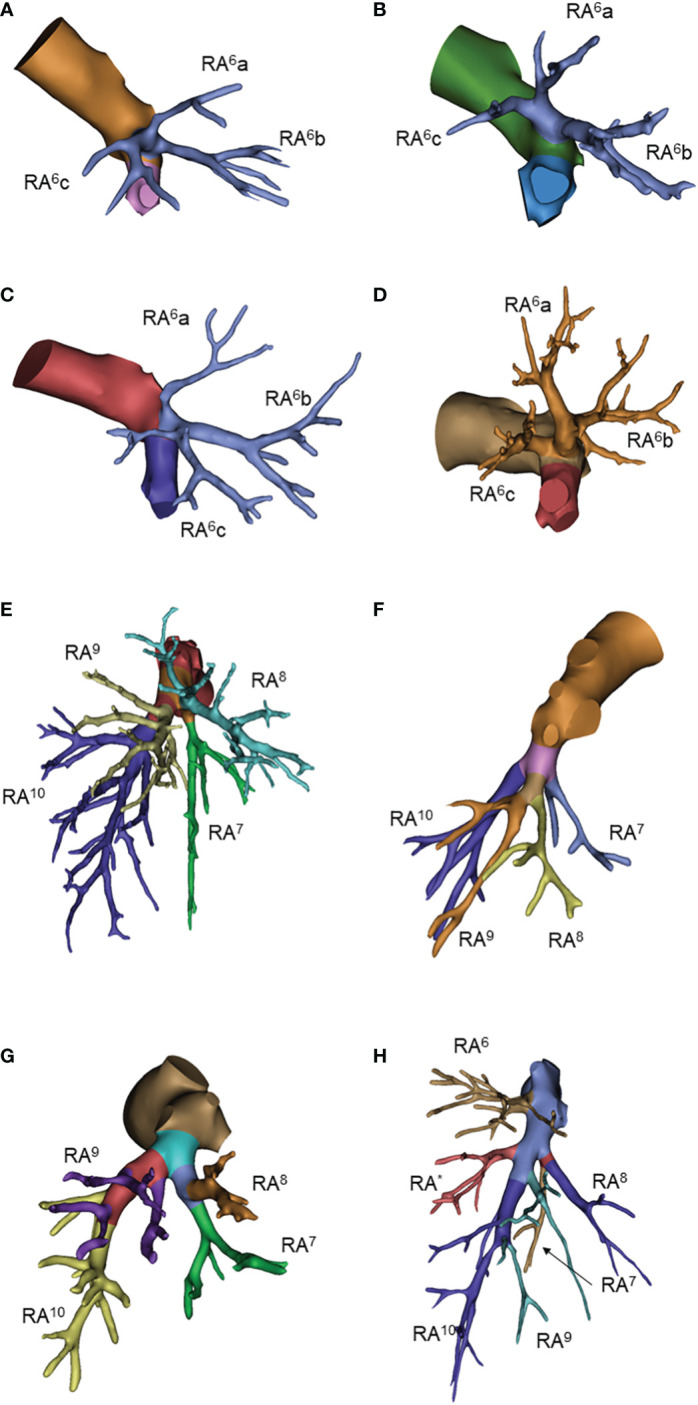
Common anatomic variants of right lower lobe. **(A)** RA6a, RA6b, RA6c; **(B)** RA6a+c, RA6b; **(C)** RA6b+c, RA6a; **(D)** RA6a+b, RA6c; **(E)** RA7, RA8, RA9+10; **(F)** RA7, RA8+9, RA10; **(G)** RA7+8, RA9+10; **(H)** RA^*^.

For the right superior segmental artery (RA^6^), we focused on the first branching point of the main trunk RA^6^. The three-sub-branch type was predominant that account for 50% cases, all of which was RA^6^a, RA^6^b and RA^6^c type. Two-sub-branch type presented as RA^6^a+b and RA^6^c, RA^6^a+c and RA^6^b, or RA^6^b+c and RA^6^a type accounted for only 50% (6%, 36% and 8%, respectively) (**Table 4**; **Figures 4A–D**, **Supplementary Figure 3**)

**Table 4:** Right lower lobe.

Artery variants	Abbreviation	Frequency
Superior segment variants※		
RA^6^a+b, RA^6^c		6%
RA^6^a+c, RA^6^b		36%
RA^6^b+c, RA^6^a		8%
RA^6^a, RA^6^b, RA^6^c		50%
Basal segment variants		
RA^7^+^8^+^9^, RA^10^	7+8+9, 10	1%
RA^7^+^9^+^10^, RA^8^	7+9+10, 8	2%
RA^7^+^8^b, RA^8^a+^9^+^10^	7+8, 8 + 9+10	2%
RA^7^+^8^, RA^9^+^10^	7+8, 9 + 10	6%
RA^7^+^8^b, RA^8^a+^9^b, RA^9^a+^10^	7+8, 8 + 9, 9 + 10	1%
RA^7^+^8^, RA^9^, RA^10^	7+8, 9, 10	1%
RA^7^, RA^8^+^9^, RA^10^	7, 8 + 9, 10	10%
RA^7^, RA^8^a+^9^+^10^, RA^8^b	7, 8, 8 + 9+10	2%
RA^7^, RA^8^, RA^9^+^10^	7, 8, 9 + 10	53%
RA^7^+^8^b, RA^8^a, RA^9^a, RA^9^b+^10^	7+8, 8, 9, 9 + 10	1%
RA^7^, RA^8^+^9^b, RA^9^a+^10^	7, 8 + 9, 9 + 10	1%
RA^7^, RA^8^+^9^a, RA^9^b+^10^	7, 8 + 9, 9 + 10	1%
RA^7^, RA^8^, RA^9^a, RA^9^b+^10^	7, 8, 9, 9 + 10	4%
RA^7^, RA^8^, RA^9^, RA^10^	7, 8, 9, 10	4%
RA^7^, RA^8^b, RA^8^a+^9^, RA^10^	7, 8, 8 + 9, 10	3%
RA^7^a, RA^8^, RA^7^b+^9^+^10^	7, 8, 7 + 9+10	3%
RA^7^+^9^a, RA^8^, RA^9^b+^10^	7+9, 8, 9 + 10	1%
RA^7^a, RA^7^b+^8^b, RA^8^a, RA^9^+^10^	7, 7 + 8, 8, 9 + 10	1%
RA^7^, RA^8^a+^9^a, RA^8^b, RA^9^b+^10^	7, 8, 8 + 9, 9 + 10	1%
RA^7^, RA^8^a, RA^8^b+^9^, RA^10^	7, 8, 8 + 9, 10	1%
RA^7^, RA^8^, RA^9^+^10^b+c, RA^10^a	7, 8, 9 + 10, 10	1%
RA^7^, RA^8^, RA^9^+^10^b+c, RA^10^a	7, 8, 9 + 10, 10	1%
RA^*^		7%

※ The first branching point of RA^6^.

The basal arteries had many branching variations. The three-branch type was observed in 67% of patients, of which RA^7^, RA^8^ and RA^9^+^10^ was the predominant pattern. The two-branch type was observed in only 11% of patients. The four-branch type has a large variation but only accounted for 23% of all patients. The right subsuperior segment artery (RA^*^) was observed in 7 patients (**Table 4**; **Figures 4E–H** and **Supplementary Figure 3**).

### Anatomical variant of the left upper lobe

**Figure 5:**
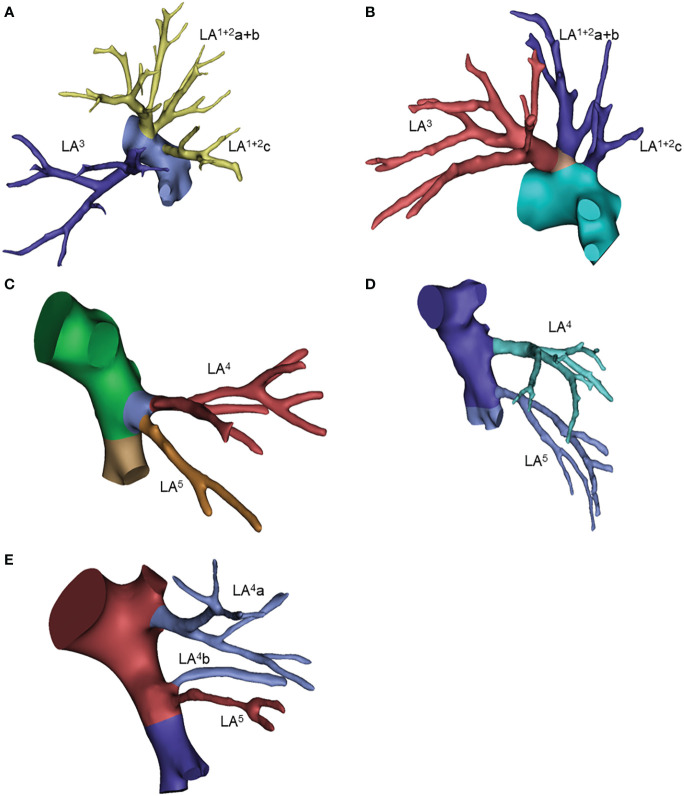
Common anatomic variants of the left upper lobe. **(A)** LA(1 + 2)a+3, LA(1 + 2)b, LA(1 + 2)c; **(B)** LA(1 + 2)a+b+3, LA(1 + 2)c; **(C)** LA4+5; **(D)** LA4, LA5; **(E)** LA4a, LA4b, LA5.

The three-branch type of superior division (S^1+2^+^3^) was observed in 53% of patients, of which the LA^1+2^a+^3^, LA^1+2^b and LA^1+2^c type accounted for 46%. The two-branch pattern was the second most common type, of which the LA^1+2^a+b+^3^ and LA^1+2^c type accounted for 35%. The single stem, four-branch and five-branch types were rare for 1%, 6% and 1%, respectively. We also noticed that LA3a branched from the intersegmental artery together with the lingual artery in 2 cases (**Table 5**; **Figures 5A, B**, **Supplementary Figure 4**).

**Table 5:** Left upper lobe.

Artery variants	Abbreviation	Frequency
LS^1+2^+^3^ artery variants		
LA^1+2^a+b+c	1+2+3	1%
LA^1+2^a+b+^3^b+c, LA^1+2^c+^3^a	1+2+3, 1 + 2+3	1%
LA^1+2^a+^3^, LA^1+2^b+c	1+2+3, 1 + 2	2%
LA^1+2^a+b+^3^, LA^1+2^c	1+2+3, 1 + 2	35%
LA^1+2^a+^3^b+c, LA^1+2^b+^3^a, LA^1+2^c	1+2+3, 1 + 2+3, 1 + 2	1%
LA^1+2^a+^3^b+c, LA^1+2^b+c, LA^3^a+^4^+^5^	1+2+3, 1 + 2, 3 + 4+5	1%
LA^1+2^a+^3^, LA^1+2^b, LA^1+2^c	1+2+3, 1 + 2, 1 + 2	46%
LA^1+2^a+b+^3^b+c, LA^1+2^c, LA^3^a+^4^b	1+2+3, 1 + 2, 3 + 4	1%
LA^1+2^a+b+^3^b+c, LA^1+2^c, LA^3^a	1+2+3, 1 + 2, 3	2%
LA^1+2^a+^3^b+c, LA^1+2^b+c, LA^3^a	1+2+3, 1 + 2, 3	1%
LA^1+2^a+^3^a, LA^1+2^b+c, LA^3^b+c	1+2+3, 1 + 2, 3	1%
LA^1+2^a+b+c, LA^3^a, LA^3^b+c	1+2, 3, 3	1%
LA^1+2^a+^3^b+c, LA^1+2^b, LA^1+2^ci, LA^1+2^cii+^3^a	1+2+3, 1 + 2, 1 + 2, 1 + 2+3	1%
LA^1+2^a+^3^b+c, LA^1+2^b, LA^1+2^c, LA^3^a	1+2+3, 1 + 2, 1 + 2, 3	3%
LA^1+2^a, LA^1+2^b+c, LA^3^a, LA^3^b+c	1+2, 1 + 2, 3, 3	1%
LA^1+2^a, LA^1+2^b, LA^1+2^c, LA^3^a, LA^3^b+c	1+2, 1 + 2, 1 + 2, 3, 3	1%
LA^1+2^a, LA^1+2^b, LA^1+2^c, LA^3^a+^4^+^5^, LA^3^b+c	1+2, 1 + 2, 1 + 2, 3 + 4+5, 3	1%
LS^4^+^5^ artery variants		
LA^3^a+^4^+^5^	3+4+5	2%
LA^4^+^5^	4+5	53%
LA^3^a+^4^b, LA^4^a+^5^	3+4, 4 + 5	1%
LA^4^a+^5^, LA^4^b	4+5, 4	2%
LA^4^+^5^bi, LA^5^a+bii	4+5, 5	1%
LA^4^+^5^a, LA^5^b	4+5, 5	4%
LA^4^, LA^5^	4, 5	29%
LA^4^a, LA^4^b, LA^5^	4, 4, 5	5%
LA^4^, LA^5^a, LA^5^b	4, 5, 5	2%
LA^4^a, LA^4^b, LA^5^a+bi, LA^5^bii	4, 4, 5, 5	1%
Mediastinal lingual artery		
LA^4^		5%
LA^4^b		5%
LA^4^+^5^a		3%
LA^4^a		2%
LA^3^a+^4^b		1%
LA^4^+^5^bi		1%
LUL artery branches from BPA		
LA^5^		6%
LA^4^, LA^5^		5%
LA^5^b		4%
LA^4^+^5^		4%
LA^4^a, LA^5^		1%
LA^5^bii		1%

BPA, basal pulmonary artery.

For the left lingular segment, common trunk LA^4^+^5^ and independent LA^4^ and LA^5^ were observed in 53% and 29% of patients, respectively. The mediastinal type of lingual artery, including the 4 types shown in the table, was observed in 17 patients (17%). In 21% of patients, lingular arteries originated from the basal artery, as shown in **Table 5** (**Figures 5C–E**, **Supplementary Figure 4**).

### Anatomical variant of the left lower lobe

**Figure 6:**
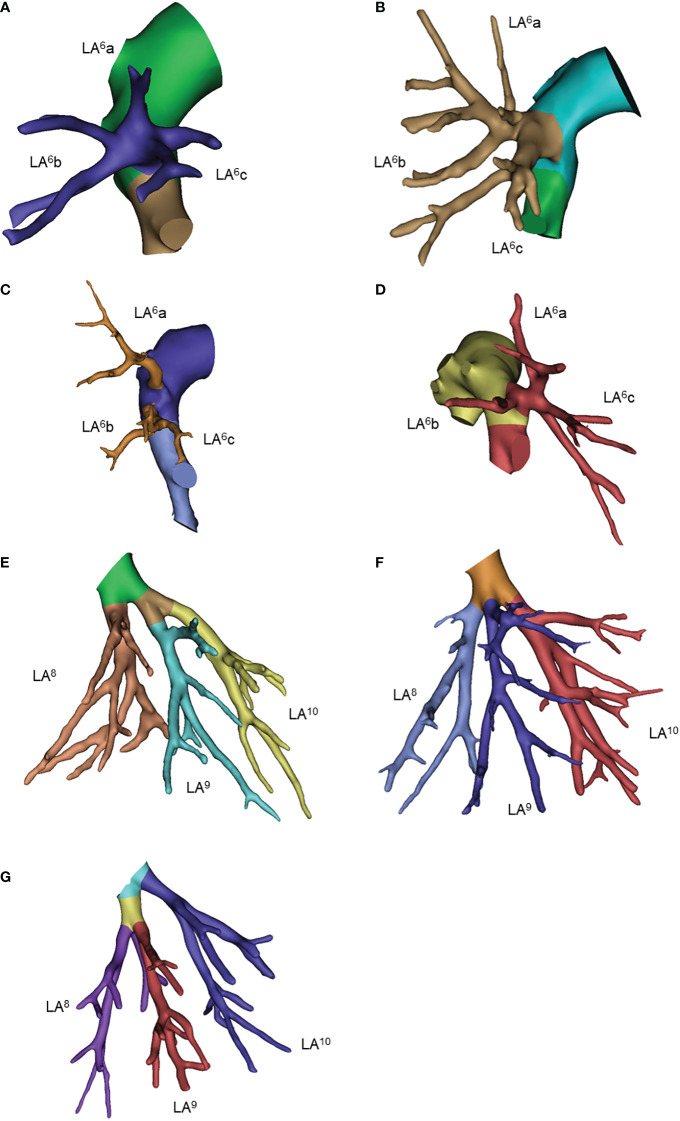
Common anatomic variants of the left lower lobe. **(A)** LA6a, LA6b, LA6c; **(B)** LA6a+b, LA6c; **(C)** LA6a, Lab+c; **(D)** LA6a+c, LA6b; **(E)** LA8, LA9+10; **(F)** LA8, LA9, LA10; **(G)** LA8+9, LA10.

For the left superior segmental artery (LA^6^), we focused on the first branching point of the main trunk LA^6^. The three-sub-branch type was the predominant type, of which the LA^6^a, LA^6^b and LA^6^c type accounted for 71%. While the two-sub-branch type presented as the LA^6^a+b and LA^6^c type, LA^6^a+c and LA^6^b type and LA^6^b+c and LA^6^a type accounted for only 28% (15%, 6% and 7%, respectively). Of note, one patient had LA^6^ and LA^9^+^10^ co-stemmed from the intersegmental artery (**Table 6**; **Figures 6A–D**, **Supplementary Figure 5**).

**Table 6:** Left lower lobe.

Artery variants	Abbreviation	Frequency
Superior segment variants※		
LA^6^a+b, LA^6^c		15%
LA^6^a+c, LA^6^b		6%
LA^6^b+c, LA^6^a		7%
LA^6^a, LA^6^b, LA^6^c		71%
LA^6^a, LA^6^b+^9^+^10^, LA^6^c		1%
Basal segment variants		
LA^8^+^9^, LA^10^	8+9, 10	17%
LA^8^a+bi, LA^8^bii+^9^+^10^	8, 8 + 9+10	1%
LA^8^, LA^9^+^10^	8, 9 + 10	40%
LA^8^+^9^a, LA^9^b+^10^	8+9, 9 + 10	3%
LA^8^+^9^ai, LA^9^aii+b+^10^	8+9, 9 + 10	1%
LA^8^+^9^ai+b, LA^9^aii+^10^	8+9, 9 + 10	1%
LA^8^+^9^a+bi, LA^9^bii+^10^	8+9, 9 + 10	1%
LA^8^, LA^6^+^9^+^10^	8, 6 + 9+10	1%
A4a+5+^8^, A^9^, A^10^	4+5+8, 9, 10	1%
LA5b+^8^, LA^9^, LA^10^	5+8, 9, 10	1%
LA5+^8^, LA^9^, LA^10^	5+8, 9, 10	1%
LA^8^a+^9^b, LA^8^b, LA^9^a+^10^	8, 8 + 9, 9 + 10	1%
LA^8^a, LA^8^b+^9^b, LA^9^a+^10^	8, 8 + 9, 9 + 10	1%
LA^8^a, LA^8^b+^9^, LA^10^	8, 8 + 9, 10	1%
LA^8^, LA^9^+^10^b+c, LA^10^a	8, 9 + 10, 10	1%
LA^8^b, LA^8^a+^10^, LA^9^b	8, 9, 8 + 10	1%
LA^8^, LA^9^a, LA^9^b+^10^	8, 9, 9 + 10	1%
LA^8^, LA^9^a+^10^, LA^9^b	8, 9, 9 + 10	1%
LA^8^, LA^9^b, LA^9^a+^10^	8, 9, 9 + 10	1%
LA^8^, LA^9^, LA^10^	8, 9, 10	23%
LA^8^a+bi, LA^8^bii+^9^b, LA^9^ai, LA^9^aii+^10^	8, 8 + 9, 9, 9 + 10	1%
LA^*^		2%

※ The first branching point of LA^6^.

Many branching variations were observed in the left basal arteries. The two-branch type was observed in 65% of patients, of which LA^8^ and LA^9^+^10^ was the predominant pattern. The three-branch type was observed in only 34% of patients. The left subsuperior segment artery (LA^*^) was observed in only 2 patients. (**Table 6**; **Figures 6E–G** and **Supplementary Figure 5**).

### A form constructed to assist in arterial identification

Based on the arterial variants we identified, a form to help surgery planning was established as shown in **Supplementary Tables 1-5**. This table includes all the variants that have a frequency > 5% that we identified, which helps to standardize the surgical planning process. It was also used as a rapid tool for assess surgery planning accuracy in another study of ours (5).

### Error recognition analysis

The overall case-wise accuracy is 35% (**Table 7**). A total of 65% of cases exhibited recognition error to some extent. On the subsegmental level, the top 10 errors are RA^4^b, LA^9^, RA^7^b, RA^1^ai, RA^9^, LA^8^a, LA^8^a+bii, RA^8^a, LA^3^a, and RA^9^a, with error rates ranging from 21 – 4% (**Supplementary Table 6**). On the segmental level, RA^4^, LA^8^, RA^9^, RA^8^, LA^9^, LA^4^, and RA^7^ showed error rates ≥ 10% (**Table 8**). At the lobular level, the recognition accuracy for the right upper lobe is 84%, right middle lobe 77%, right lower lobe 50%, left upper lobe 82%, and left lower lobe 67% (**Table 7**).

**Table 7:** Lobular recognition accuracy.

Lobe	Accuracy
Left upper lobe	82%
Left lower lobe	67%
Right upper lobe	84%
Right middle lobe	77%
Right lower lobe	50%
Overall	35%

**Table 8:** Segmental error compilation.

Ground truth branches	Mislabeled branches (%)	Error rate
LA^1+2^	LA^4^ (1)	1%
LA^3^	LA^1+2^ (3) LA^4^ (1)	4%
LA^4^	LA^5^ (10)	10%
LA^5^	LA^4^ (1) LA^8^ (2)	3%
LA^6^		0
LA^8^	LA^9^ (17)	17%
LA^9^	LA^8^ (2) LA^10^ (9)	11%
LA^10^	La^9^ (5)	5%
LA*	LA^9^ (1) LA^10^ (1)	2%
RA^1^	RA^2^ (8) RA^3^ (1)	9%
RA^2^	RA^1^ (2) RA^3^ (2)	4%
RA^3^	RA^2^ (3)	3%
RA^4^	RA^5^ (22) RA^8^ (1)	23%
RA^5^		0
RA^6^		0
RA*	RA^9^ (1) RA^10^ (1)	2%
RA^7^	RA^8^ (2) RA^10^ (8)	10%
RA^8^	RA^8^ (2) RA^9^ (12)	14%
RA^9^	RA^9^ (3) RA^10^ (14)	17%
RA^10^	RA^7^ (1) RA^9^ (8)	9%

## Discussion

In this study, we present a comprehensive pulmonary artery atlas on a sub-subsegmental level to aid lobectomy and segmentectomy and provide a rapid tool form for surgery planning. We have also analyzed all errors during the data labeling process to demonstrate the spectrum of common misidentifications as a resource for thoracic surgeons.

We noticed that most frequencies of anatomic variants in our study corroborated previous publications (**Supplementary Table 7**), except for RA^1^ and bilateral basal segments. Two major reasons were responsible for such differences.

First, the criterion of common trunk is difficult to define. From the imaging perspective, directly adjacent RA^1^a and RA^1^b or a short distance between the branching point of two arteries are difficult to judge. From a surgical perspective, a short common trunk may still demonstrate as 2 separated arteries due to intraoperative traction of the lung. To evade this confusing scenario, we used the most rigid criterion for common trunk, and only strictly separated branches that have no bordering pixels on the 3-D model should be considered independent. Despite the difference in whether RA1a is independent or not, all RA^1^a was the first branch from the pulmonary artery, which will not cause any surgical confusion.

Second, the subsegmental structure of the bilateral basal segment is more complicated than expected. Only 67% of right and 83% of left basal segmental artery branches according to Norami’s categories (11). Twenty-three percent of right and 16% of left basal segmental arteries branch on the sub-segmental or more distal level. The same trend was also observed in LA^3^, and right middle lobe arteries. Such findings should raise enough attention during basal segmentectomy, since arteries and bronchi may branch on different levels, and potential mis-ligation may be avoided through cautious planning. While for right middle lobe, since RS^4^ or RS^5^ segmentectomy is rarely reported, the difference of branching point between different 2-branch subtypes may be less important for surgery purpose.

To our surprise, only 4% of right pulmonary arteries and as many as 21% of left pulmonary arteries showed cross-lobular variations. All cross-lobular variations occurred between the middle lobe (including left lingular segment) and lower lobe (**Table 3**, **5**). RA^2^ branches from RA^6^ (or vice versa) was reported to be relatively frequently seen (12), but it was not observed in our study cohort due to the limited case number.

Another major surgical concern is mediastinal type lingular arteries. In our study, the total frequency of mediastinal type artery is 17%, corroborating other studies. However, none of the mediastinal arteries is consisted of both LA^4^ and LA^5^. It is also worth noting that division from the subsegmental level is not rarely seen in lingular arteries and is all related to either mediastinal type or cross-lobular variations.

In the error analysis, the average segmental artery labeling accuracy is 91%. In our previous publications, the recognition accuracies of segmental arteries are 54.9% and 59.6%. This may be due to the difference in recognition time consumption. Our previous study was conducted under a clinical practice scenario, and the attending tended to finish the recognition task as fast as possible, which rendered the average recognition time at 120 seconds. In this study, the purpose is to obtain labels that are as accurate as possible. The slow labeling process also allows surgeons to go back and forth for correction, which gives rise to the relatively higher accuracy.

The most frequent recognition error was observed in RA^4^b, which is natural due to the rare surgical application of RS^4^ or RS^5^ resection. Basal segmental arteries also showed a high frequency of mis-identification due to unexpected branching points, as we have pointed out. Confusion between RA^7^ and RA^10^, RA^8^ and RA^9^, LA^9^ and LA^10^ are mostly seen. In some publications (13–15), absent basal segmental arteries such as A^7^ or A^9^ were reported but not well defined. For A^7^ absent, we found it extremely rare and did not encounter any case with a suspicious absent A^7^ yet. A^9^ was suspected to be absent in a couple of cases, while they were finally ruled out using the existence of intersegmental veins (V^8^b and V^9^b). In our study, we were generally more preservative on declaring the absent of a certain segment, which may also give rise to a slightly higher error rate on basal segments.

Most recognition errors were intra-lobular, of which the confusion appeared between adjacent segments. Most errors were concentrated in the bilateral basal segments, especially in subsegmental arteries. We should notice that more than 1/5 of basal segmental arteries branch on subsegmental or even more distal level; we also noticed a high confusion rate between subsegmental RA^2^ and RA^1^, which made arbitrary intra-operative ligation of arteries during resections for basal segments, RS^1^ and RS^2^ highly risky for collateral damage.

Cross-lobular error (taken cross-segmental error between LS^1+2^+^3^ and LS^4^+^5^ into consideration as well) was seen in 5% of cases, 2% between LS^1+2^+^3^ and LS^4^+^5^, 2% between LS^4^+^5^ and the left lower lobe, and 1% between right middle lobe and right lower lobe. LA^4^, LA^8^ and RA^8^ are most likely to be mis-identified as other lobular arteries, which should be more cautiously prepared before surgery.

The accuracy of automated vessel reconstruction algorithm of chest CT has been a lasting enthusiasm in both academia and industry thanks to the development of deep learning. Nardelli etc. has proved the technical feasibility (16), Wang etc. (17) and Li etc. (18) also provided clinically feasible algorithms. From the regulation prospect, multiple three-dimensional reconstruction software is actively applying for approval indicating a foreseeable future of universal clinical deployment of such algorithm. Our study group has also moved one step further into automated semantic segmentation of 3D reconstruction images (5) aiming to name each segmental arteries for better surgery support. By fulfilling the machine cognition of pulmonary anatomy, future applications such as surgery route suggestion, rare anatomical variation warning, and even automated or semi-automated surgery might become possible.

Our study is limited by the number of cases. Rare anatomical variants are under-represented, although they have significant value for surgical planning. A cohort of rare anatomic variants is being prospectively collecting for further study.

To summarize, our research provided an atlas for lobectomy and segmentectomy at the subsegmental or even more distal level and suggested a quick surgery planning tool accordingly. To our knowledge, we are the first to demonstrate that the recognition accuracy of pulmonary arteries in a non-time-sensitive experimental scenario was still unfavorable. We also suggested that certain segments (S^1^, S^2^ and basal segments) should receive extra caution during surgical planning process.

## Data availability statement

The raw data supporting the conclusions of this article will be made available by the authors, without undue reservation.

## Ethics statement

The studies involving human participants were reviewed and approved by Institutional Review Board of Peking University People’s Hospital (2022PHB011-002). Written informed consent for participation was not required for this study in accordance with the national legislation and the institutional requirements.

## Author contributions

HX, HZ, JJ, XC are responsible for data labeling. HX, HZ and XC drafted the manuscript. JG participated in data analysis. CS and NH are responsible for data collection. DW is responsible for 3D reconstruction of DICOM files. FY and XC are responsible for manuscript alterations. All authors contributed to the article and approved the submitted version.
