# Supplementary material for: An atlas of anatomical variants of subsegmental pulmonary arteries and recognition error analysis
Source: Front Oncol. 2023 Mar 13;13:1127138. doi: 10.3389/fonc.2023.1127138 (PMC10040796; doi:10.3389/fonc.2023.1127138)
Supplement: Supplementary file 1 [file Presentation_1.pptx]

## Slide 1
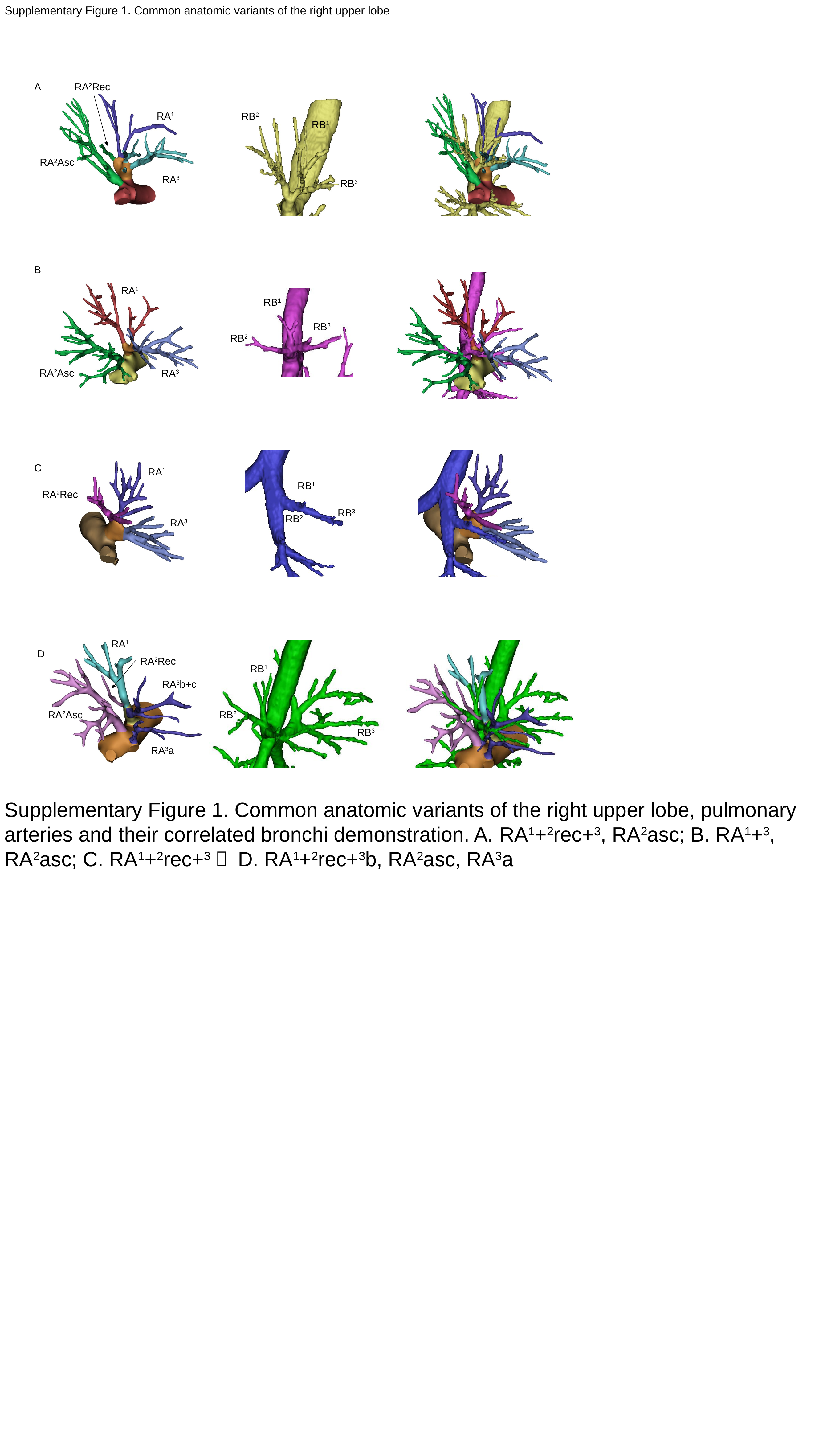

# Supplementary Figure 1. Common anatomic variants of the right upper lobe
A
RA2Rec
RA1
RB2
RB1
RA2Asc
RA3
RB3
B
RA1
RB1
RB3
RB2
RA2Asc
RA3
C
RA1
RB1
RA2Rec
RB3
RB2
RA3
RA1
D
RA2Rec
RB1
RA3b+c
RA2Asc
RB2
RB3
RA3a
Supplementary Figure 1. Common anatomic variants of the right upper lobe, pulmonary arteries and their correlated bronchi demonstration. A. RA1+2rec+3, RA2asc; B. RA1+3, RA2asc; C. RA1+2rec+3； D. RA1+2rec+3b, RA2asc, RA3a

## Slide 2
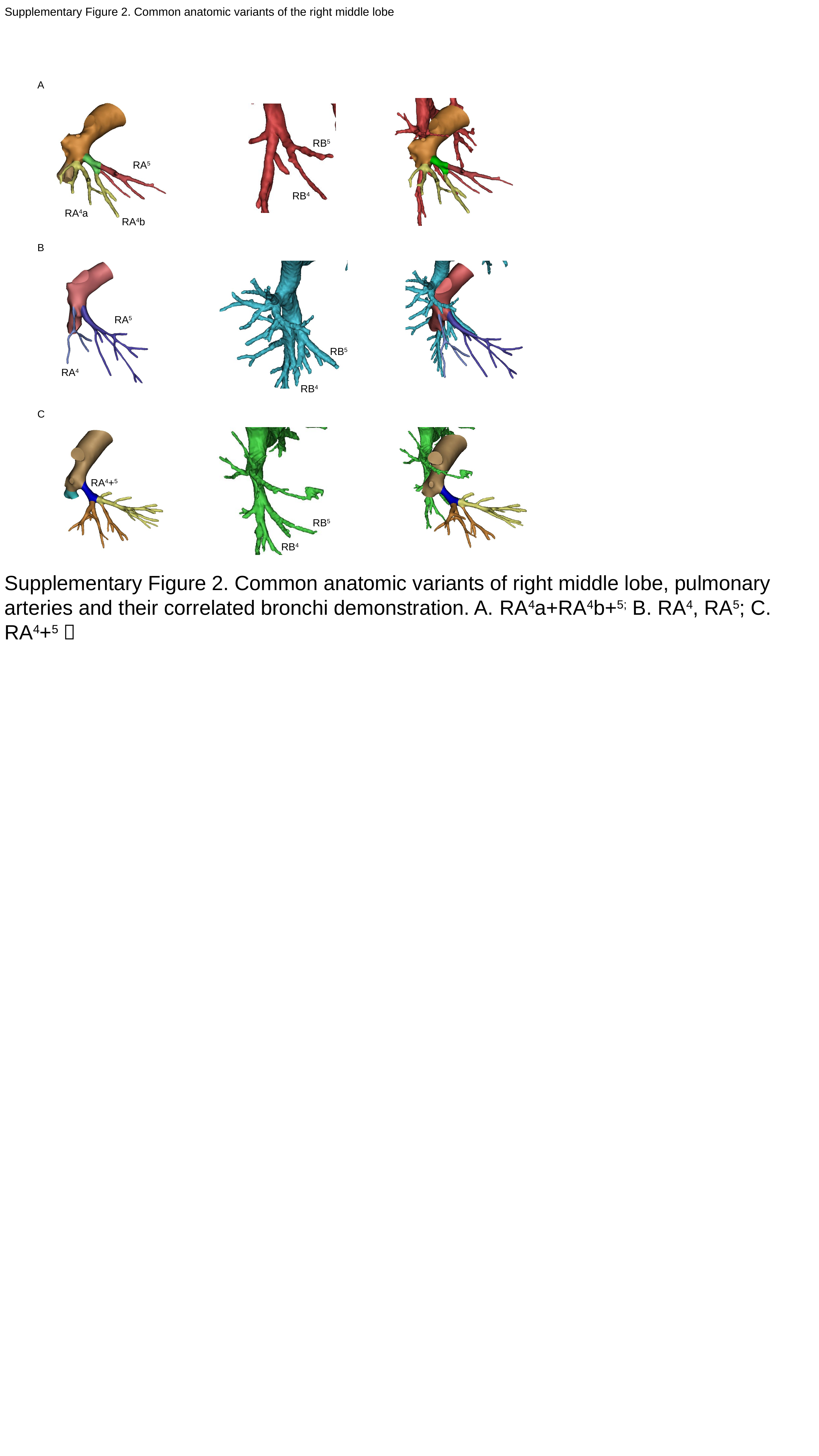

# Supplementary Figure 2. Common anatomic variants of the right middle lobe
A
RB5
RA5
RB4
RA4a
RA4b
B
RA5
RB5
RA4
RB4
C
RA4+5
RB5
RB4
Supplementary Figure 2. Common anatomic variants of right middle lobe, pulmonary arteries and their correlated bronchi demonstration. A. RA4a+RA4b+5; B. RA4, RA5; C. RA4+5；

## Slide 3
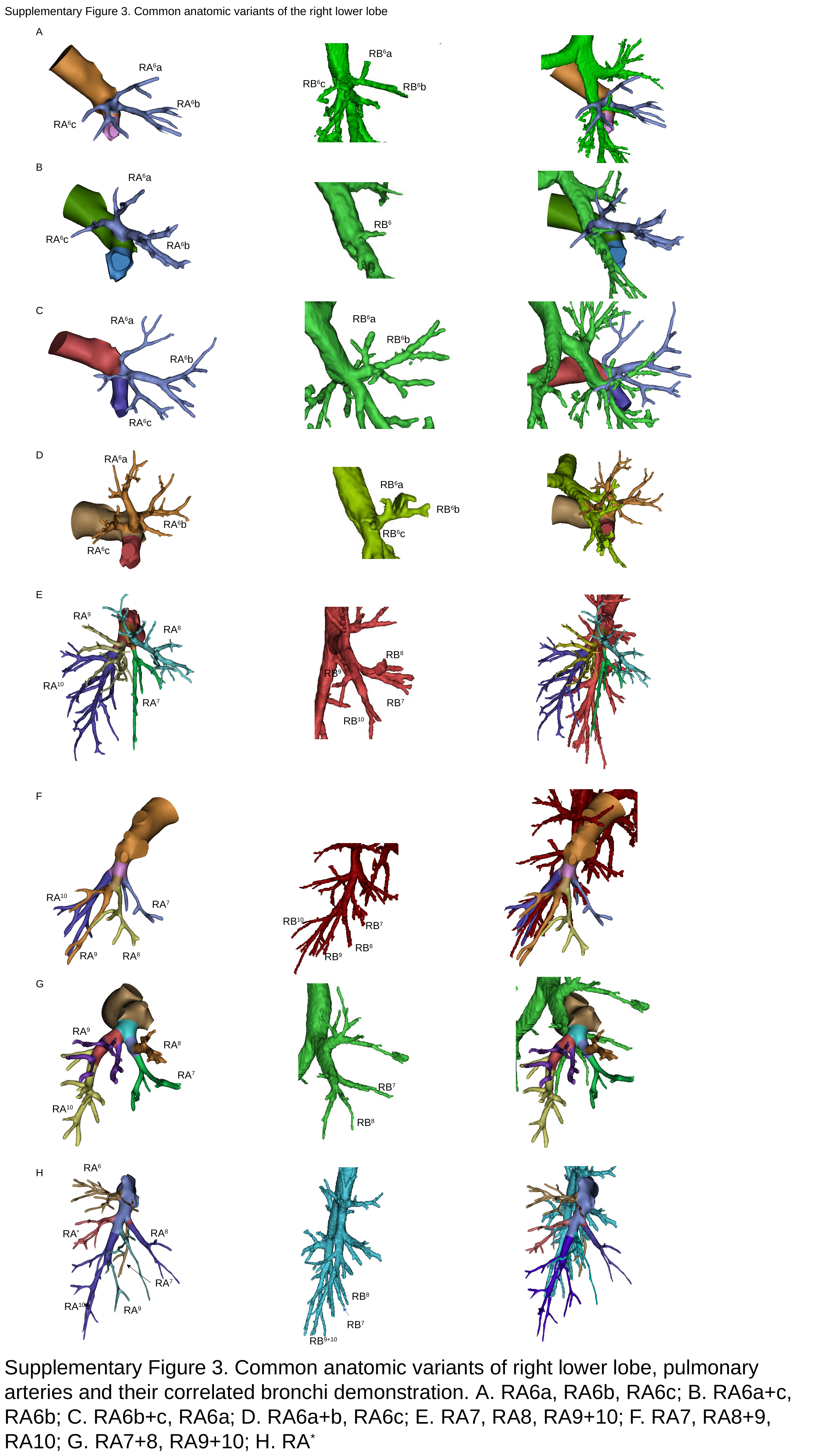

# Supplementary Figure 3. Common anatomic variants of the right lower lobe
A
RB6a
RA6a
RB6c
RB6b
RA6b
RA6c
B
RA6a
RB6
RA6c
RA6b
C
RB6a
RA6a
RB6b
RA6b
RA6c
D
RA6a
RB6a
RB6b
RA6b
RB6c
RA6c
E
RA9
RA8
RB8
RB9
RA10
RA7
RB7
RB10
F
RA10
RA7
RB10
RB7
RB8
RA9
RA8
RB9
G
RA9
RA8
RA7
RB7
RA10
RB8
RA6
H
RA8
RA*
RA7
RB8
RA10
RA9
RB7
RB9+10
Supplementary Figure 3. Common anatomic variants of right lower lobe, pulmonary arteries and their correlated bronchi demonstration. A. RA6a, RA6b, RA6c; B. RA6a+c, RA6b; C. RA6b+c, RA6a; D. RA6a+b, RA6c; E. RA7, RA8, RA9+10; F. RA7, RA8+9, RA10; G. RA7+8, RA9+10; H. RA*

## Slide 4
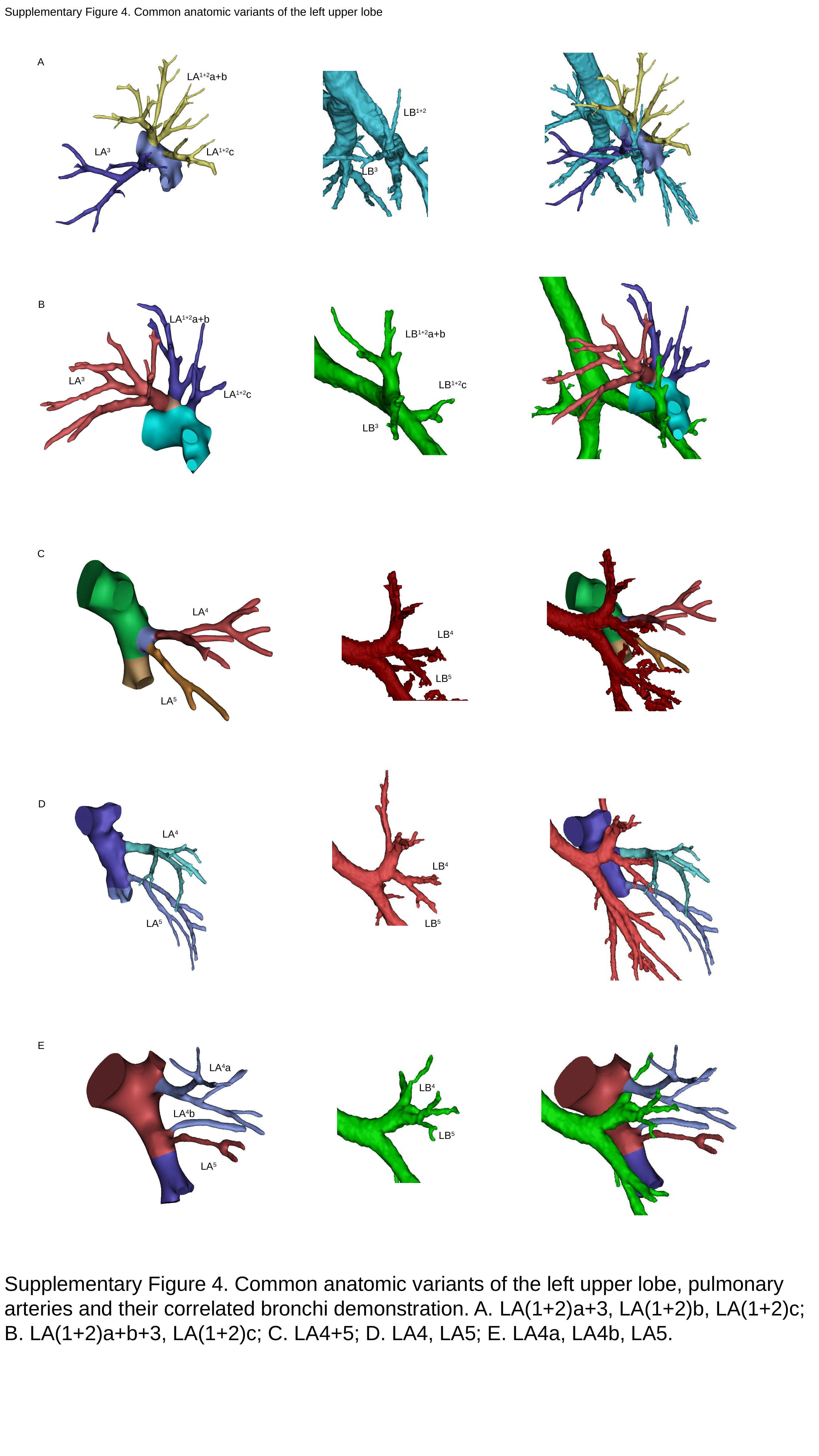

# Supplementary Figure 4. Common anatomic variants of the left upper lobe
A
LA1+2a+b
LB1+2
LA3
LA1+2c
LB3
B
LA1+2a+b
LB1+2a+b
LA3
LB1+2c
LA1+2c
LB3
C
LA4
LB4
LB5
LA5
D
LA4
LB4
LB5
LA5
E
LA4a
LB4
LA4b
LB5
LA5
Supplementary Figure 4. Common anatomic variants of the left upper lobe, pulmonary arteries and their correlated bronchi demonstration. A. LA(1+2)a+3, LA(1+2)b, LA(1+2)c; B. LA(1+2)a+b+3, LA(1+2)c; C. LA4+5; D. LA4, LA5; E. LA4a, LA4b, LA5.
LA5

## Slide 5
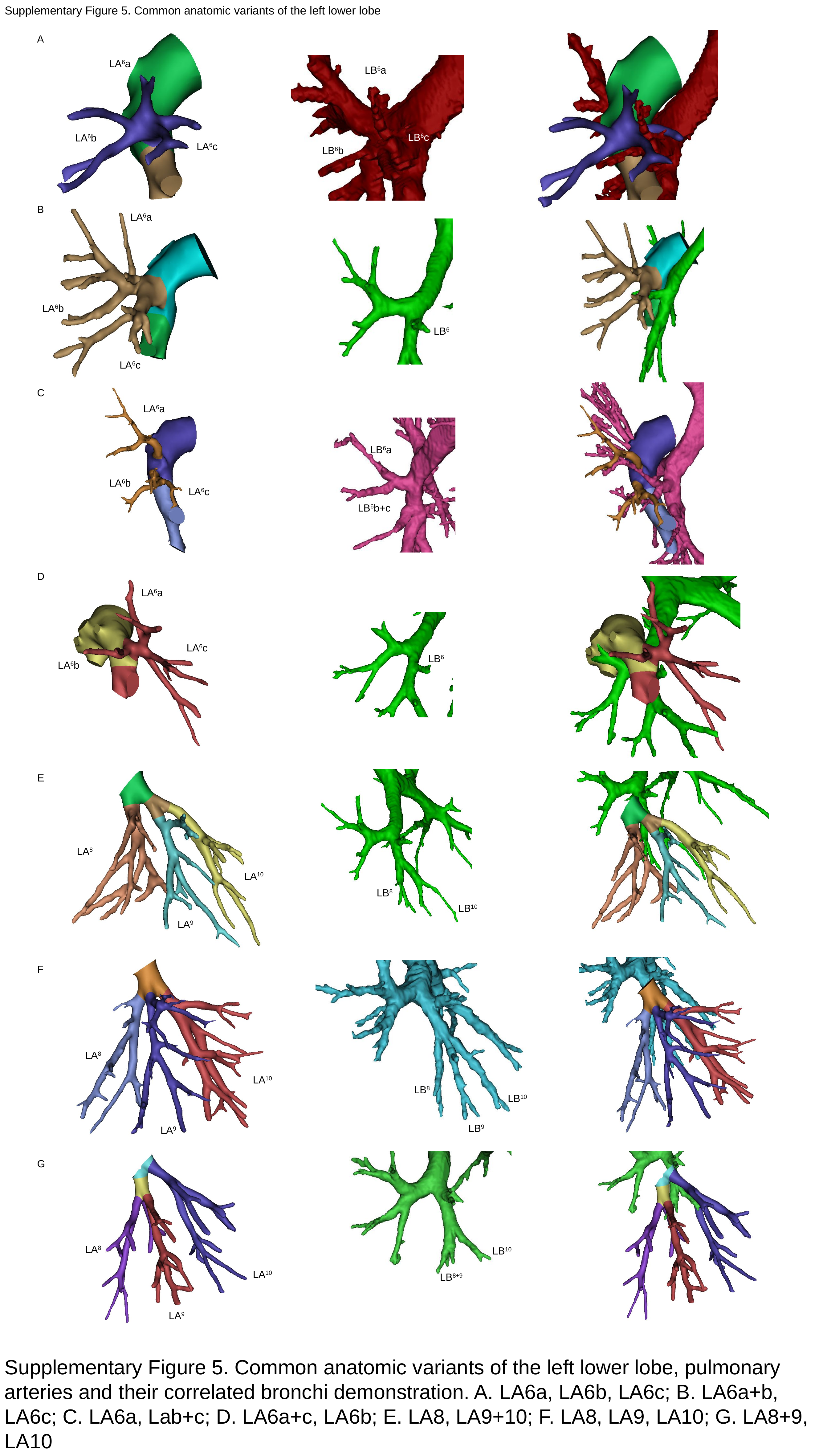

# Supplementary Figure 5. Common anatomic variants of the left lower lobe
A
LA6a
LB6a
LB6c
LA6b
LA6c
LB6b
B
LA6a
LA6b
LB6
LA6c
C
LA6a
LB6a
LA6b
LA6c
LB6b+c
D
LA6a
LA6c
LB6
LA6b
E
LA8
LA10
LB8
LB10
LA9
F
LA8
LA10
LB8
LB10
LB9
LA9
G
LA8
LB10
LA10
LB8+9
LA9
Supplementary Figure 5. Common anatomic variants of the left lower lobe, pulmonary arteries and their correlated bronchi demonstration. A. LA6a, LA6b, LA6c; B. LA6a+b, LA6c; C. LA6a, Lab+c; D. LA6a+c, LA6b; E. LA8, LA9+10; F. LA8, LA9, LA10; G. LA8+9, LA10
