# Supplementary material for: An atlas of anatomical variants of subsegmental pulmonary arteries and recognition error analysis
Source: Front Oncol. 2023 Mar 13;13:1127138. doi: 10.3389/fonc.2023.1127138 (PMC10040796; doi:10.3389/fonc.2023.1127138)
Supplement: Supplementary file 2 [file Table_1.docx]

**Supplementary table 1. Right upper lobe surgery planning form**

| **Right Upper Lobe** | **Artery** | | | |
| --- | --- | --- | --- | --- |
|  | **lobular variants** | **A1** | **A2** | **A3** |
| **Type 1** | No inter-lobular variant | Both A1a and A1b from truncus superior artery | A2a from recurrent artery; A2b from ascending artery | Both A3a and A3b from truncus superior artery |
| **Type 2** | A2 from A6 | A1a branches independently, A1b from truncus superior artery | Both A2a and A2b from ascending artery | A3a from truncus intermedius; A3b from truncus superior artery |
| **Type 3** | Other | Other | Both A2a and A2b from recurrent artery | A3a from truncus superior; A3b from truncus intermedius |
| **Type 4** |  |  | Other | Other |
| **Type 5** |  |  |  |  |
| **Type 6** |  |  |  |  |

**Supplementary table 2. Right middle lobe surgery planning form**

| **Right Middle Lobe** | **Artery** | |
| --- | --- | --- |
|  | **lobular variants** | **A4+5** |
| **Type 1** | A4 and A5 share a same truncus | Branches from main pulmonary artery |
| **Type 2** | A4 and A5 are independent | A4 branches from basal artery, A5 branches from main pulmonary artery |
| **Type 3** | Other | A5 branches from basal artery, A4 branches from main pulmonary artery |
| **Type 4** |  | Other |
| **Type 5** |  |  |
| **Type 6** |  |  |

**Supplementary table 3. Right lower lobe surgery planning form**

| **Right Lower Lobe** | **Artery** | | | | | | |
| --- | --- | --- | --- | --- | --- | --- | --- |
|  | **lobular variants** | | **A6** | **A7** | **A8** | **A9** | **A10** |
| **Type 1** | A* | No inter-lobular variant | One branch | Forms common truncus with A8 | Forms common truncus with A7 | Forms common truncus with A10 | Forms common truncus with A9 |
| **Type 2** | Absent A* | A6 from A2 | Two branches | Branches from basal pulmonary artery | Forms common truncus with A9 | Forms common truncus with A8 | A10 |
| **Type 3** |  | A7 from middle lobe artery | Three branches | Absent A7 | Independent | Independent | Independent |
| **Type 4** |  | Other | Other | Independent | Other | Other | Other |
| **Type 5** |  |  |  | Other |  |  |  |
| **Type 6** |  |  |  |  |  |  |  |

**Supplementary table 4. Left upper lobe surgery planning form**

| **Left Upper Lobe** | **Artery** | | | | | |
| --- | --- | --- | --- | --- | --- | --- |
|  | **lobular variants** | **A1+2** | **A1+2d** | **A3** | **A4** | **A5** |
| **Type 1** | No inter-lobular variant | A1+2a+b and A1+2c | Absent | A3 branches from main pulmonary artery | A4 branch from interlobar artery | A5 branch from interlobar artery |
| **Type 2** | A2 from A6 | A1+2a, A1+2b, and A1+2c | One | A3b+c branches from main pulmonary artery, and A3a branches distal to A1+2c | A4+5 branches from interlobar artery | A4+5 branches from interlobar artery |
| **Type 3** | A4+5 from basal artery | A1+2a and A1+2b+c | Other | Other | A4+5 branches from mediastinum (main pulmonary artery) and runs between V1+3 and B3 | A4+5 branches from mediastinum (main pulmonary artery) and runs between V1+3 and B3 |
| **Type 4** | Other | A1+2a+b+c |  |  | A4 branches from mediastinum (main pulmonary artery) and runs between V1+3 and B3 | A5 branches from mediastinum (main pulmonary artery) and runs between V1+3 and B3 |
| **Type 5** |  | Other |  |  | Other | Other |
| **Type 6** |  |  |  |  |  |  |

**Supplementary table 5. Left lower lobe surgery planning form**

| **Left Lower Lobe** | **Artery** | | | | | |
| --- | --- | --- | --- | --- | --- | --- |
|  | **lobular variants** | | **A6** | **A8** | **A9** | **A10** |
| **Type 1** | A* | No inter-lobular variant | One branch | Independent | Forms common truncus with A10 | Forms common truncus with A9 |
| **Type 2** | Absent A* | A6 from A2 | Two branches | Forms common truncus with A9 | Forms common truncus with A8 | Independent |
| **Type 3** |  | Other | Other | Other | Independent | Other |
| **Type 4** |  |  |  |  | Other |  |
| **Type 5** |  |  |  |  |  |  |
| **Type 6** |  |  |  |  |  |  |

| **Supplementary table 6: Sub-segmental error compilation** | | |
| --- | --- | --- |
| **Ground truth branches** | **Mislabeled branches (%)** | **Error rate** |
| LA^1+2^b | LA^4^ (1) | 1% |
| LA^3^a | LA^1+2^ (3)  LA^4^ (1) | 4% |
| LA^4^a | LA^5^ (3) | 3% |
| LA^4^ai/ii | LA^5^ (3) | 3% |
| LA^4^b | LA^5^ (3) | 3% |
| LA^4^bi | LA^5^ (1) | 1% |
| LA^5^a | LA^4^ (1) | 1% |
| LA^5^bii | LA^8^ (2) | 2% |
| LA^8^a | LA^9^ (5) | 5% |
| LA^8^a+bii | LA^9^ (5) | 5% |
| LA^8^aii | LA^9^ (3) | 3% |
| LA^8^aii+bii | LA^9^ (1) | 1% |
| LA^8^b | LA^9^ (1) | 1% |
| LA^8^bii | LA^9^ (2) | 2% |
| LA^9^ | LA^10^ (8) | 8% |
| LA^9^b | LA^8^ (2)  LA^10^ (1) | 3% |
| LA^10^a | LA^9^ (1) | 1% |
| LA^10^a+b | LA^9^ (2) | 2% |
| LA^10^b | LA^9^ (2) | 2% |
| LA^*^ | LA^9^ (1)  LA^10^ (1) | 2% |
| RA^1^ai | RA^2^ (7) | 7% |
| RA^1^bi | RA^2^ (1)  RA^3^ (1) | 2% |
| RA^2^a | RA^1^ (1) | 1% |
| RA^2^ai | RA^1^ (1) | 1% |
| RA^2^b | RA^3^ (2) | 2% |
| RA^3^a | RA^2^ (3) | 3% |
| RA^4^ai | RA^8^ (1) | 1% |
| RA^4^b | RA^5^ (21) | 21% |
| RA^4^bi | RA^5^ (1) | 1% |
| RA* | RA^9^ (1)  RA^10^ (1) | 2% |
| RA^7^ | RA^8^ (1) | 1% |
| RA^7^a | RA^8^ (1) | 1% |
| RA^7^b | RA^10^ (8) | 8% |
| RA^8^ | RA^9^ (2) | 2% |
| RA^8^a | RA^9^ (5) | 5% |
| RA^8^ai/ii | RA^9^ (3) | 3% |
| RA^8^aii+bii | RA^9^ (1) | 1% |
| RA^8^bi | RA^7^ (2) | 2% |
| RA^8^bii | RA^9^ (1) | 1% |
| RA^9^ | RA^8^ (1)  RA^10^ (6) | 7% |
| RA^9^a | RA^8^ (2)  RA^10^ (2) | 4% |
| RA^9^ai+b | RA^10^ (1) | 1% |
| RA^9^aii | RA^10^ (2) | 2% |
| RA^9^b | RA^10^ (2) | 2% |
| RA^9^bii | RA^10^ (1) | 1% |
| RA^10^ | RA^7^ (1)  RA^9^ (2) | 3% |
| RA^10^a+b | RA^9^ (2) | 2% |
| RA^10^b | RA^9^ (3) | 3% |
| RA^10^b+c | RA^9^ (1) | 1% |

| **Supplementary Table 7: Comparison between published data** | | | |
| --- | --- | --- | --- |
|  | Our result | Norami | Others |
| **RA1** |  |  |  |
| RA1a+b branches from truncus superior artery | 100 | 68 |  |
| RA1a branches independently | 0 | 32 |  |
| **RA2** |  |  | ref(13) |
| RA2 recurrent and ascending both exist | 53 | 72 |  |
| RA2 recurrent only | 22 | 12 | 10 |
| RA2 ascending only | 25 | 16 |  |
| **RA3** |  |  | (13) |
| RA3a+b branches from truncus superior artery | 75 | 48 |  |
| RA3a from truncus intermedius, RA3b from truncus superior artery | 16 | 34 | 38 |
| RA3a from truncus superior, RA3b from truncus intermedius | 7 | 18 |  |
| Other RA3 variants | 2 | 0 |  |
| **RA4 and RA5** |  |  | ref(13) |
| RA4+5 branches from truncus intermidius | 21 |  | 36 |
| RA4, RA5 branch from truncus intermidius | 31 |  | 62 |
| RA4a, RA4b+5 branches from truncus intermidius | 41 |  |  |
| Other RA4 and RA5 variants | 7 |  | 2 |
| **RA7** |  |  | ref(13) |
| RA7+8 branches from basal pulmonary artery | 7 | 60 | 18 |
| RA7 branches from basal pulmonary artery | 81 | 24 | 66 |
| RA7 absent | 0 | 16 | 0 |
| Other RA7 variants | 12 | 0 | 16 |
| **RA8-10** |  |  | ref(13) |
| RA8, RA9+10 | 53 | 90 | 70 |
| RA8+9, RA10 | 10 | 8 | 14 |
| RA8, RA9, RA10 | 4 | 2 | 0 |
| Other RA8-10 variants | 33 | 0 | 16 |
| **RA*** |  |  |  |
| RA* exist | 7 | 4 |  |
| RA* absent | 93 | 96 |  |
| **LA(1+2)** |  |  | ref(7) |
| LA(1+2)a+b, LA(1+2)c | 39 | 31 | 32 |
| LA(1+2)a, LA(1+2)b, LA(1+2)c | 52 | 28 | 34 |
| LA(1+2)a, LA(1+2)b+c | 6 | 26 | 20 |
| LA(1+2)a+b+c | 2 | 15 | 14 |
| Other LA(1+2) variants | 1 | 0 | 0 |
| **LA3** |  |  |  |
| LA3a, LA3b+c branch from truncus superior | 84 | 90 |  |
| LA3b+c branches from truncus superior, LA3a branches from truncus intermidius | 11 | 10 |  |
| Other LA3 variants | 5 | 0 |  |
| **LA4 and LA5** |  |  | ref(7) |
| Mediastinal type LA4+5 | 0 | 18 | 7 |
| Mediastinal and interlobar type | 17 | 12 | 17 |
| Interlobar type | 83 | 70 | 79 |
| LA4, LA5 branch from interlobar artery | 24 | 26 |  |
| LA4+5 branches from interlobar artery | 55 | 44 |  |
| Other interlobar type | 4 | 0 |  |
| **LA8-10** |  |  | ref(15) |
| LA8, LA9+10 | 40 | 74 | 52 |
| LA8+9, LA10 | 17 | 16 | 42 |
| LA8, LA9, LA10 | 26 | 10 | 3 |
| Other LA8-10 variants | 17 | 0 | 3 |
